# Rituximab-Induced Kaposi Sarcoma in HIV-Negative Patients: A Narrative Review

**DOI:** 10.7759/cureus.45365

**Published:** 2023-09-16

**Authors:** Alexandru Oprita, Horia Cotan, Dana Celmare, Radu Emilescu

**Affiliations:** 1 Oncology, Carol Davila University of Medicine and Pharmacy, Bucharest, ROU; 2 Oncology, Saint Nicholas Hospital, Pitesti, ROU; 3 Oncology, Elias Emergency University Hospital, Bucharest, ROU; 4 Oncology, Saint Nicholas Medical Center, Pitesti, ROU; 5 Endocrinology, Diabetes and Metabolism, C.I. Parhon National Institute of Endocrinology, Bucharest, ROU

**Keywords:** iatrogenic kaposi sarcoma, kaposi sarcoma, kaposi sarcoma management, metachronous cancer, rituximab

## Abstract

Kaposi sarcoma (KS) is a low-grade mesenchymal angioproliferative disorder that requires infection with human herpes virus 8 (HHV-8) for it to develop. It is commonly seen in HIV-positive patients and rarely in immunosuppressed HIV-negative patients. Rituximab is a monoclonal anti-CD20 chimeric murine/human immunoglobulin G antibody used to treat B cell lymphoproliferative diseases as well as a variety of autoimmune disorders. Several cases of iatrogenic Kaposi sarcoma (iKS) have been described after rituximab treatment.

The purpose of this narrative review is to identify the presence of common clinical characteristics among rituximab-induced KS patients that could facilitate better management of this rare condition.

## Introduction and background

Kaposi sarcoma (KS) was described for the first time in 1872 by Moritz Kaposi. He observed several cases of cutaneous multifocal pigmented sarcoma [1] and named them idiopathic multiple-pigmented sarcoma of the skin. Low-grade mesenchymal angioproliferative disorders like KS most frequently affect immunocompromised people like HIV-positive patients and organ transplant recipients [2]. Human herpesvirus type 8 (HHV-8/KS-associated herpesvirus) lytic replication is the primary cause of KS. Primary effusion lymphoma [3], HIV-associated multicentric Castleman's disease (HIV-MCD) [4], and the newly described Kaposi sarcoma inflammatory cytokine syndrome [5] have also been linked to the pathophysiology of HHV-8. The prevalence of KS among HIV patients has significantly decreased since the introduction of antiretroviral therapy [6], indicating that patient immunological status is a key predictor of KS development.

Currently, there are four clinical variants of KS, each with distinct natural history, prognosis, and site propensity: (i) classic KS mainly affects the extremities of the lower limbs of elderly men in Mediterranean countries and the Middle East; (ii) the epidemic subtype is associated with acquired immunodeficiency syndrome (AIDS-associated KS), which mainly affects men who have sex with men infected with the human immunodeficiency virus (HIV); (iii) iatrogenic subtype occurs in patients treated with immunosuppressive therapy, especially organ transplant recipients; (iv) endemic subtype is observed primarily in sub-Saharan Africans [7]. Iatrogenic KS (iKS) is the term used to describe KS brought on by the use of steroids, immunosuppressive drugs, and agents with anti-tumor necrosis factor (TNF) activity in patients with autoimmune diseases, inflammatory diseases, or solid organ transplantation [8,9]. The iatrogenic variant of KS is rarely reported and accounts for 5% to 20% of the total number of reported cases [10]. Owing to immune suppression by cancer or chemotherapy, cancer patients are also prone to KS.

Rituximab is a monoclonal anti-CD20 chimeric murine/human immunoglobulin G antibody used to treat B cell lymphoproliferative disease [11]. Its mechanism of action consists of CD20+ B cell depletion through complement-dependent and antibody-dependent cell-mediated cytotoxicity. Rituximab is also administered in a variety of autoimmune diseases such as rheumatoid arthritis, systemic lupus erythematosus, antineutrophil cytoplasmic antibody-associated granulomatous vasculitis, refractory nephrotic syndrome, and humoral organ transplant rejection [12-14].

Rituximab therapy has been reported to exacerbate HIV-associated KS, most likely by immune system suppression in already immunocompromised HIV patients. To date, rituximab-related KS in HIV-negative patients has been reported by only a few articles in the literature. Rituximab may thus have a role in HHV-8 lytic replication and facilitate the development of KS in the absence of HIV infection. The goal of this narrative review is to provide an overview of the role of rituximab in the development of KS in HIV-negative patients (KS-nHIV).

## Review

Methods

**Figure 1:**
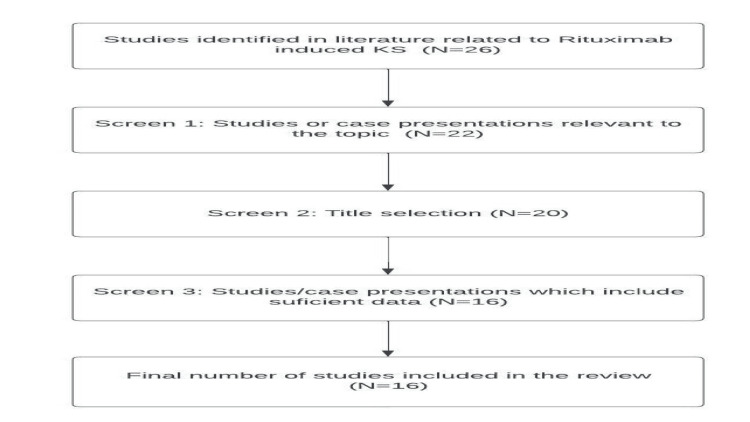
Flow diagram for study selection

A literature search evaluating the role of rituximab in the development of KS was conducted using the National Library of Medicine (MEDLINE), the Excerpta Medica database (EMBASE), and the Cochrane Database of Systematic Reviews (CDSR). The literature search was conducted between May 2023 and June 2023. The inclusion criteria consisted of English-language articles, case series, and case studies that reported on the role of rituximab in the development of KS. We examined the title and abstract of each identified study, and the full text of the study was consulted for relevant studies. The following keywords were used in the search: Kaposi sarcoma, iatrogenic Kaposi sarcoma, and rituximab-induced Kaposi sarcoma. A flowchart describing article selection is included in Figure 1.

Pathogenesis of KS

HHV-8, commonly known as Kaposi sarcoma-associated herpes virus (KSHV), is a major contributor to the pathogenesis of KS [15,16]. Chang et al. first discovered the DNA virus known as KSHV in a KS lesion of an AIDS patient in 1994 [17]. KSHV is believed to be the infectious agent required for the development of all clinical subtypes of KS, irrespective of variations in presentation, natural history, or prognosis [18]. The discovery that KSHV generates inflammatory and angiogenic cytokines, as well as gene products linked to angiogenesis, directly links this virus to the pathophysiology of KS [19]. There are three main clinical stages of KS skin lesions: (i) Patch stage: flat lesions, not raised above the surrounding skin, (ii) Plaque stage: the lesions are flat but slightly raised, (iii) Nodule stage: The lesions turn into bumps on the skin, known as nodules [20]. The viral load of the KSHV correlates with the clinical progression of KS from patch to plaque and finally to the nodule stage [18]. The role of KSHV in the development of KS is further supported by higher incidence in KSHV-positive solid organ transplant patients and HIV-positive patients [21]. KSHV may not be enough for the emergence and progress of KS, although its presence is necessary. This could help to explain why KSHV infection is usually asymptomatic and does not result in KS despite the high incidence of the virus in high-risk populations and endemic regions. The regression of iKS after cessation of immunosuppressive therapy further suggests KSHV as a necessary but insufficient cofactor in KS development.

The role of HIV and KSHV coinfection in the development of KS cannot be understated [22]. For example, in men with both HIV and KSHV, the risk of developing KS appears to rise by 60% for every year of HIV infection, and these viruses likely work in concert to cause KS [18]. It is hypothesized that HIV-mediated immune dysregulation/suppression encourages the production of T-helper type-1 cytokines such as TNF-alpha, IL-1b, and IL-6. These cytokines facilitate a chronic state of inflammation which is conducive to the development and progression of KS [18,23]. Furthermore, the production and release of HIV-TaT protein mediate the reactivation and replication of latent KSHV. The development of KS in HIV-seronegative patients shows the involvement of HIV gene products to not be essential in all KS forms. Although incomplete, the HIV-KSHV coinfection model suggests the significant role of immunosuppression in the pathogenesis of KS [24].

Role of rituximab in KS development

Rituximab is the first monoclonal antibody drug used in oncology. It was approved by the Food and Drug Administration in 1997. The first licensed indication was for the treatment of relapsed/refractory indolent non-Hodgkin lymphoma. Since its approval, rituximab’s therapeutic indications were extended and presently include numerous other B-cell malignancies, autoimmune diseases, inflammatory disease, paraneoplastic pemphigus, pemphigus vulgaris, hypocomplementemic urticarial vasculitis, autoimmune cytopenias, and humoral organ transplant rejection. Rituximab is also prescribed for multicentric Castleman’s disease and primary effusion lymphoma [11,25].

Rituximab is a human/mouse chimeric monoclonal antibody (IgG1) that targets the CD20 antigen expressed in more than 95% of normal and malignant B cells and induces complement-mediated and antibody-dependent cellular cytotoxicity. It has been reported as a triggering factor for KS [26]. Rituximab depletes B cells and causes a decrease in T-cell activation because of decreased antigen presentation by B cells. The formation of autoantibodies against T cells is an additional cellular immunity depletion mechanism. Rituximab causes reactivation of hepatitis B and thus prophylactic antiviral therapy is recommended [26].

The main targets of HHV-8 are B cells and endothelial cells, thus B cells, due to their long life, can be major latent reservoirs of HHV-8. Activated CD4+ T cells are capable of suppressing virus production by these infected B cells. In contrast, a major decrease in the number of CD4+ T cells is considered a significant risk factor associated with KS and multicentric Castleman’s disease, whatever the cause of the T cell-dependent immunosuppression [27].

Lower B cell counts were also associated with a statistically significant increased risk of KS development, while higher B cell counts were protective against KS development. These data highlight a potential role for B cells and the humoral immune system in KS pathogenesis [28]

**Figure 2:**
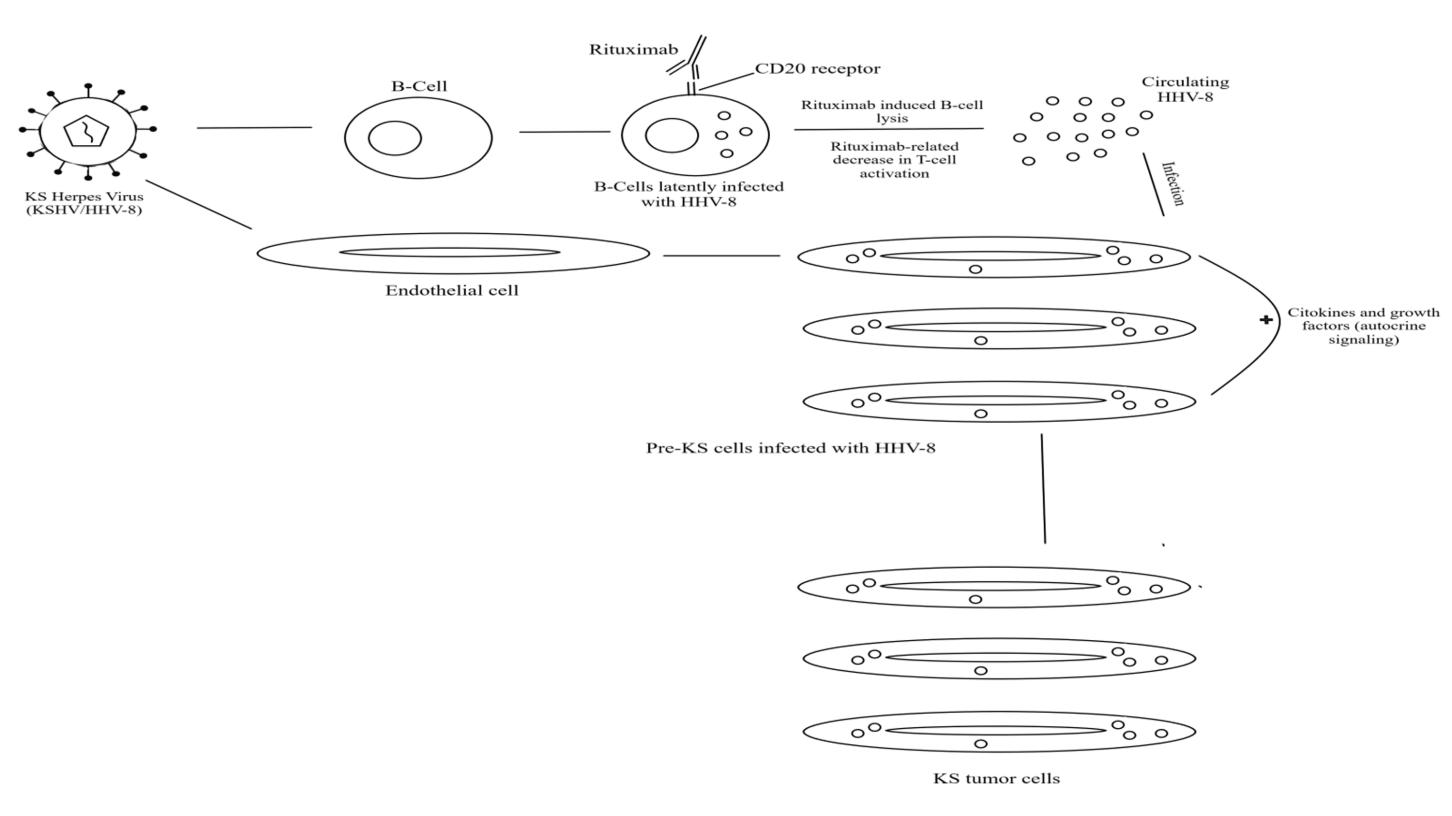
Role of rituximab in the development of KS in HIV-negative patients

Some authors believe that a rituximab-related decrease in T-cell activation plays a key role in HHV8 reactivation. Although rituximab was a groundbreaking drug in the treatment of B-cell lymphomas, rituximab-associated activation of hepatitis B and HHV-8 are adverse effects that delay chemotherapy. The risk-benefit ratio of rituximab, especially for non-lymphomatous disorders, such as autoimmune and systemic inflammatory disorders, needs to be carefully assessed for patients at risk of developing HHV-8 tumors [27]. The role of rituximab in the pathogenesis of KS is showcased in Figure 2.

Rituximab-induced KS in patients treated for non-Hodgkin lymphoma

The number of cases of patients developing KS after treatment with rituximab for hematologic malignancies is limited in number, consisting mainly of case reports.

A case study by Billon et al. reports on a KS case that developed under rituximab treatment for follicular lymphoma in an HIV-negative, 55-year-old patient [29]. The lesion developed at the level of the rectum, approximately one year after rituximab initiation, more precisely during maintenance treatment. No cutaneous lesions were observed. After cessation of rituximab, the KS completely disappeared without the need for any specific therapy.

Another case report by Alkan et al. describes the case of a 65-year-old treated for stage IIA B cell lymphoma with an R-CHOP regimen (rituximab 375 mg/m^2^, vincristine 2 mg, cyclophosphamide 750 mg/m^2^ on day 1, doxorubicin 50 mg/m^2^ on day 1 and methylprednisolone 80 mg for five days, every three weeks) [26]. After 15 weeks of R-CHOP, the patient presented several purple cutaneous macular lesions on the upper and lower limbs that were later confirmed as KS. Nuclear positivity for HHV-8 was detected. After discontinuation of rituximab, the lesions were treated with radiotherapy. The patient was in remission for B-cell lymphoma and showed no signs of KS at two years follow-up.

Ureshino et al. described a similar case in an older HIV-negative patient with numerous comorbidities who was treated with an R-CHOP regimen for diffuse large B cell lymphoma (DLBCL) [30]. After complete remission of the DLBCL (21 weeks from R-CHOP induction), the patient developed purple plaques on the left foot which later spread to the right arm and genital area. Lesions were confirmed as HHV-8 negative KS. All KS lesions were resected and at 17 months after R-CHOP induction the patient remains in complete remission for both DLBCL and KS.

Another case is that of a 52-year-old male patient with a history of mantle cell lymphoma treated with rituximab, dexamethasone, cytarabine, cisplatin/oxaliplatin (R-DHA-oxaliplatin/cisplatin), and autologous stem-cell transplantation. Five months after treatment initiation the patient noticed several cutaneous lesions on the nasal alae and ear lobes. Skin biopsy confirmed the KS diagnosis and tumor cell nuclei stained positive for HHV-8. Rituximab treatment was stopped and the patient remained in follow-up. After one year, the KS lesions have remained stable [31].

A case report by Uyanik et al. describes the case of a 69-year-old male patient with splenic marginal zone lymphoma treated with R-CHOP regimen with partial response [32]. The patient displayed recurrence 18 months after R-CHOP induction, with the administration of RFC (rituximab, fludarabine, and cyclophosphamide) as second-line therapy. Thirty months after rituximab induction, the patient developed multiple purple/blue cutaneous lesions on both legs, which were confirmed as KS via biopsy. After stopping rituximab, vinblastine 2mg/m^2^ was initiated for KS. The KS lesions exhibited partial response after two months of vinblastine therapy; however, the patient later died due to complications unrelated to KS or lymphoma.

Rituximab-induced KS in patients treated for chronic lymphocytic leukemia

A case report by Belur et al. showcases the case of a 76-year-old HIV-negative female patient diagnosed with chronic lymphocytic leukemia (CLL) [33]. The patient was treated with six cycles of day 1 rituximab 375mg/m^2^ and day 1 and day 2 bendamustine 60mg/m^2^. After 18 weeks of rituximab treatment, the patient developed a series of papular lesions on both lower extremities. After the biopsy, the diagnosis was established as KS with positive HHV-8 staining. Doxorubicin 20mg/m^2^ was initiated and within three cycles there was a significant lesion response.

A retrospective study by Brambilla et al. included 1389 KS patients of which 143 were suffering from iKS [34]. Patients with iKS were divided into two research arms consisting of patients who received organ transplants (OTR) and those who were immunosuppressed for other reasons. OTR developed iKS at a younger age (71.9 yrs non-OTR vs. 51.4 yrs OTR, p=0.004) but non-OTR patients exhibited a more severe disease at diagnosis compared to OTR patients (stage IVb: 29.1% vs. 12.1%, p=0.001). The study mentions the inclusion of two patients treated with rituximab (RTX) associated with bendamustine and prednisone for chronic lymphatic leukemia in the non-OTR group.

Similarly, another case report describes the case of a 70-year-old male patient diagnosed with CLL and treated with six cycles of rituximab + fludarabine + cyclophosphamide. After a short interval, a purple lesion appeared on the left foot of the patient. The diagnosis of HHV-8-positive KS was established following the biopsy. The patient underwent radiation at a total dose of 30 Gy with the disappearance of the skin lesion after three months [35].

Rituximab-induced KS in organ transplant recipients

As seen in the previous section, organ transplant recipients (OTR) had a higher chance of developing KS compared to patients who were immunosuppressed for other reasons.

A retrospective study by Périer et al. included 988 patients that received rituximab and only four male HIV-negative, HHV-8 tumor-positive patients developed KS, at a median of four months after the first RTX infusion [27]. One 56-year-old patient was treated with rituximab for a heart transplant after failure to achieve immunosuppression with other agents. He developed KS four months after rituximab induction and was treated with radiotherapy and bleomycin, followed by doxorubicin after progression. The patient did not respond favorably to treatment and died 20 months after rituximab induction.

 A 2010 retrospective study by Scemla et al. reported on the complications associated with the use of rituximab in renal transplant patients [36]. Out of 38 patients included in this study, two developed KS. Both patients were previously treated with various immunosuppressive medications without success.

Rituximab-induced KS in patients with autoimmune disease

KS was described in several cases of autoimmune disease treated with rituximab. A case report describes a 52-year-old male HIV-negative patient diagnosed with thrombotic thrombocytopenic purpura (TTP) that was initially treated with corticosteroids. After initial improvement in platelet count, he exhibited severe thrombocytopenia that was treated with four weekly doses of rituximab at 800 mg per dose. After therapy completion, he developed several purple lesions on his upper and lower extremities. These lesions were diagnosed as KS and treated conservatively with liquid nitrogen cryo-ablation. At follow-up, the patient had no recurrence of TTP or KS [37].

Another report by Daflaoui et al. describes the case of an 82-year-old male with pemphigus vegetans that was treated with a total of two doses of rituximab of 1000mg every two weeks [38]. Three months after the first rituximab dose the patient developed erythematous papules, plaques, and nodules on the right leg, left ankle, hips, and trunk. Skin biopsy from one of the lesions showed characteristics compatible with KS. The patient received treatment with topical 5-fluorouracil (5-FU), with marked resolution of the skin lesions after three months of daily 5-FU application. Total remission of KS and pemphigus vegetans was reported on follow-up.

Similarly, a case report by Torretta et al. describes the case of a 68-year-old male patient with pemphigus vulgaris who was treated with rituximab as well as corticosteroids and immunoglobulins [39]. After completion of treatment, he developed laryngeal KS which caused severe dyspnea and respiratory distress. He underwent an emergency tracheotomy which allowed the recovery of airway patency and was later treated with bleomycin. One month after surgery he developed Acinetobacter Baumannii pneumonia and subsequent septic shock which proved fatal.

Another case of rituximab-induced KS was reported in a 76-year-old female patient with stage IVb (according to the Myasthenia Gravis Foundation of America staging) myasthenia gravis that associated bulbar and respiratory manifestations. After first-line therapy with pyridostigmine and glucocorticoids which proved ineffective, rituximab treatment was proposed. After serology testing, the possibility of acute or chronic HBV, HCV, and HIV infection was excluded. Six months after rituximab induction, several purple/blue skin nodules appeared on the trunk, arms, and legs and were later confirmed as KS. Rituximab was stopped. After staging the work-up using thoracic-abdominal-pelvic computed tomography, several suspect lesions were identified in both lungs. The patient was diagnosed with stage IV KS and started on pegylated liposomal doxorubicin (PLD). Complete resolution of the nodular skin lesions was observed after the first two PLD administrations. Following six cycles of PLD, disease progression was noted and, due to poor performance status, chemotherapy was stopped and the best supportive care was initiated. The patient died 14 months after rituximab initiation [40].

 In the final case described in the literature, a 79-year-old man diagnosed with autoimmune hemolytic anemia secondary to chronic lymphocytic leukemia received treatment with methylprednisolone, which proved ineffective. Treatment with rituximab 375mg/m^2^ was proposed and after the second infusion, a complete response was observed. Before the last dose of rituximab, the patient developed nodular lesions on both legs. After a skin biopsy, the diagnosis of KS was established followed by three courses of oral etoposide 60mg/m^2^. After completion of etoposide treatment, the lesions disappeared and no recurrence was noted at follow-up [41].

Management of rituximab-induced KS

Even though no widely accepted guidelines on the management of iKS exist to date, reducing or discontinuing immunosuppressants represents the most accredited therapeutic option in iKS [34]. This therapeutic measure is often accompanied by local or systemic therapies depending on the disease stage and severity [10]. After the cessation of immunosuppressive drugs, that may have induced iKS, different therapeutic options include both local and systemic treatment (Table 1). Another employed option, described in the literature, is watchful waiting when KS is limited to the skin.

**Table 1:** Published cases of rituximab-induced iatrogenic Kaposi sarcoma in HIV-negative patients KS: Kaposi sarcoma

First author	Year	Sex	Age at diagnosis	Clinical conditions	KS localization	Immunosuppression therapy	Tumor HHV-8 status	Blood HHV-8 status	KS management
Billon et al. [29]	2018	Male	55 years	Follicular lymphoma	Rectal mucosa	Rituximab and bendamustine	HHV-8 positive	HHV-8 negative	Rituximab cessation
Alkan et al. [26]	2019	Male	65 years	Diffuse large B-cell lymphoma	Extremities	R-CHOP	HHV-8 positive	Unknown	Rituximab cessation and radiotherapy
Ureshino et al. [30]	2015	Male	84 years	Diffuse large B-cell lymphoma	Extremities and genital area	R-CHOP	HHV-8 negative	Unknown	Rituximab cessation and KS excision
Geller et al. [31]	2018	Male	52 years	Mantle cell lymphoma	Cutaneous lesions on the nasal alae and ear lobes	Rituximab, dexamethasone, cytarabine, cisplatin/oxaliplatin (R-DHA-oxaliplatin/cisplatin)	HHV-8 positive	Unknown	Rituximab cessation
Uyanik et al. [32]	2016	Male	69 years	Splenic marginal zone lymphoma	Cutaneous lesions on both legs	R-CHOP and subsequent treatment with RFC (rituximab, fludarabine, and cyclophosphamide)	Unknown	HHV-8 negative	Rituximab cessation and vinorelbine
Belur et al. [33]	2014	Female	72 years	Chronic lymphocytic leukemia (CLL)	Papular lesions on both lower extremities	Rituximab and bendamustine	HHV-8 Positive	Unknown	Rituximab cessation and doxorubicin
Hacioglu et al. [35]	2015	Male	70 years	Chronic lymphocytic leukemia (CLL)	Purple lesion on the left foot	RFC (rituximab, fludarabine, and cyclophosphamide)	HHV-8-positive	Unknown	Rituximab cessation and radiotherapy
Jerdan et al. [37]	2017	Male	52 years	Thrombotic thrombocytopenic purpura (TTP)	Several lesions on his upper and lower extremities	Corticotherapy and subsequent treatment with rituximab	HHV-8-positive	HHV-8 negative	Rituximab cessation and local therapy with liquid nitrogen cryo-ablation
Daflaoui et al. [38]	2022	Male	82 years	Pemphigus vegetans	Erythematous papules, plaques, and nodules on the right leg, left ankle, hips, and trunk	High dose Rituximab	HHV-8-positive	HHV-8 positive	Rituximab cessation and topical 5-fluorouracil (5-FU)
Torretta et al. [39]	2013	Male	68 years	Pemphigus Vulgaris	Laryngeal KS	Rituximab, corticosteroids and immunoglobulins	HHV-8-positive	Unknown	Rituximab cessation and bleomycin
Sánchez-Escribano et al. [40]	2016	Female	76 years	Myasthenia gravis	Skin nodules on the trunk, arms, and legs	Pyridostigmine and glucocorticoids and subsequent treatment with rituximab	HHV-8-positive	Unknown	Rituximab cessation and liposomal doxorubicin
Itar et al. [41]	2019	Male	79 years	Autoimmune hemolytic anemia secondary to chronic lymphocytic leukemia	Nodular lesions on both legs	Methylprednisolone and subsequent treatment with rituximab	Unknown	Unknown	Rituximab cessation and oral etoposide

Localized treatment may consist of surgical excision, radiotherapy, intralesional chemotherapy, silver nitrate cauterization, and compressive therapy with elastic stockings. Systemic treatment can consist of various drugs: liposomal doxorubicin, sirolimus (for transplant KS), paclitaxel, pomalidomide, bortezomib, gemcitabine, lenalidomide, vinorelbine, etoposide, imatinib, ipilimumab + nivolumab, pembrolizumab.

The mechanisms of rituximab-induced KS are largely unknown. Rituximab-induced B-lymphocyte depletion determines latent HHV-8 reactivation and implicitly favors the development and progression of KS [42]. Secondary to the depletion of HHV-8-infected B cells susceptible endothelial cells are exposed to higher levels of HHV-8 [43]. There is evidence, albeit limited, of the efficacy of treatment with valganciclovir. It was demonstrated that treatment with valganciclovir can reduce HHV-8 viral load and that valganciclovir can be used in high-risk patients to prevent KS flare [25]. Prophylactic treatment with valganciclovir can be administered to patients at high risk of developing KS.

Although lytic viral replication may play a role in some aspects of KS, antiviral therapy is not likely to be of benefit when used as monotherapy for KS, as the role of persistent viral replication after malignant transformation is unclear [44]. We speculate the use of adjuvant antiviral therapy targeted to HHV-8 could be a possible choice for rituximab-induced KS as could prophylactic therapy in high-risk patients.

Discussion

This narrative review aims to establish if rituximab can be responsible for KS development. While data in the literature are scarce and mostly consist of case reports several pertinent conclusions can still be made. The series of case reports included in this review describe KS occurring in individuals who have been treated with rituximab, particularly when rituximab is used in combination with other immunosuppressive therapies. This fact raises the question of whether rituximab alone can be considered a risk factor for KS development. To answer this, we must look back at the mechanism of rituximab-induced KS.

The depletion of B-cells by rituximab can weaken the immune system's ability to control viral infections, including HHV-8, which is a key factor in the development of KS [42]. Additionally, the decrease of HHV-8-infected B cells caused by rituximab may increase the exposure of HHV-8-vulnerable endothelium cells to higher amounts of HHV-8 [43].

It is essential to note that rituximab itself does not directly cause KS. Instead, it may increase the risk of developing KS in individuals who are already infected with HHV-8 or who have other risk factors, such as a compromised immune system from either a different cancer or in HIV-positive patients from acquired immunodeficiency syndrome. Therefore, patients receiving rituximab and other immunosuppressive therapies should be carefully monitored for signs of KS and other opportunistic infections, especially if they have underlying conditions that make them more susceptible to these complications.

Despite the extensive use of rituximab in the treatment of a variety of ailments, the reported case number of secondary KS in HIV-negative patients is extremely low. To our knowledge, approximately 30 cases have been described so far in the literature [45] (Table 1). While the overall risk of developing rituximab-induced iKS is quite low, a series of common clinical characteristics can be observed among these patients. Most patients were male and had been treated with multiple immunosuppressive agents alongside rituximab, corticosteroids being the most common. Although most patients were treated with multiple immunosuppressive agents, the case described by Dafloui et al. was treated with high-dose rituximab further suggesting the role of rituximab in iKS development [38]. Opportunistic infections cause systemic inflammation which may promote KS development and progression [45]. An opportunistic lung infection was described by Ureshino et al. [30] right before the development of KS. Another potential risk factor observed was age, all patients included in this review were older than 50 years (52-82 years). It is possible that among HIV-negative patients age-related immunosuppression was associated with the development of iKS as opposed to HIV-positive patients which develop KS at a younger age (<40 years) [46,47]. We also observed that severely immunosuppressed patients exhibited a more aggressive form of KS [34].

The average time from rituximab initiation until KS development was 6.8 months (1.5-30 months). All patients benefited from complete immunosuppressive therapy or just rituximab therapy cessation. With no clear guidelines for iKS management, the subsequent KS treatment varied widely, from complete resection of the lesions to systemic therapy and radiotherapy. That being said prognosis is quite favorable irrespective of treatment with most patients exhibiting complete or partial response during follow-up. Only one patient which was included in this review died during follow-up with an overall survival of 11 months.

Even though iKS is a rare occurrence, caution is required, especially for cancer patients who are frequently immunocompromised by both their condition and their treatment. That being said some key aspects of managing rituximab-induced KS in HIV-negative patients are: 1) discontinuation of rituximab, 2) KS-specific treatment (the choice of treatment depends on the extent and severity of the KS lesions), 3) management of immunosuppression (if rituximab was administered as part of a treatment regimen for an autoimmune disease, the management of immunosuppression therapy may be necessary), 4) regular monitoring (especially for patients at risk for HHV8 infection which include patients with high Fitzpatrick skin phototype, Mediterranean or equatorial African geographic origin, male gender, homosexuality or multiple sexual partners and immune deficiency. Patients at risk should be monitored for HCV (hepatitis C) and HBV (hepatitis B) infection during rituximab treatment as well as other potential opportunistic infections, and 5) preventive measures (preventing infections and complications is crucial for individuals with weakened immune systems. This may involve prophylactic antibiotics or antiviral medications, as well as lifestyle changes to minimize infection risks).

Several limitations of this review include the lack of data available in the literature, and most of the articles described being case reports. We believe that high-quality studies performed on larger patient populations should be conducted so that clear guidelines can be developed. That being said it is important to emphasize that the management of rituximab-induced KS is complex and should be tailored to the individual patient's specific medical history and condition. 

## Conclusions

Our review highlights a noteworthy association between rituximab therapy and the development of KS in HIV-negative patients. While rituximab is an effective treatment for various medical conditions, including autoimmune diseases and certain malignancies, it appears to be a potential risk factor for KS. The role of rituximab-induced immunosuppression in the pathogenesis of KS cannot be overlooked, although data is limited and only several studies report on this association. This review also suggests that not all patients receiving rituximab are at equal risk for KS. Certain patient-specific factors, such as underlying medical conditions (other cancers, autoimmune disease) and previous immunosuppressive therapies, may contribute to varying susceptibility. Timely diagnosis and monitoring of KS are paramount, especially in patients undergoing rituximab treatment. Close clinical surveillance and vigilant evaluation of skin lesions, oral mucosa, and other potential sites of KS lesions are crucial for early intervention. 

We have also shown that the management of rituximab-induced KS involves a multifaceted approach, including discontinuation of rituximab, KS-specific treatment modalities, and tailored adjustments to immunosuppressive regimens for autoimmune conditions.
