# Correction: Rituximab-Induced Kaposi Sarcoma in HIV-Negative Patients: A Narrative Review

**DOI:** 10.7759/cureus.c284

**Published:** 2025-09-08

**Authors:** Alexandru Oprita, Horia Cotan, Dana Celmare, Radu Emilescu

**Affiliations:** 1 Oncology, Carol Davila University of Medicine and Pharmacy, Bucharest, ROU; 2 Oncology, Saint Nicholas Hospital, Pitesti, ROU; 3 Oncology, Elias Emergency University Hospital, Bucharest, ROU; 4 Oncology, Saint Nicholas Medical Center, Pitesti, ROU; 5 Endocrinology, Diabetes and Metabolism, C.I. Parhon National Institute of Endocrinology, Bucharest, ROU

This article has been revised to add the following affiliation for Alexandru Vlad Oprita: Oncology, Carol Davila University of Medicine and Pharmacy, Bucharest, Romania.

